# Mass incarceration and public health: the association between black jail incarceration and adverse birth outcomes among black women in Louisiana

**DOI:** 10.1186/s12884-019-2690-z

**Published:** 2019-12-27

**Authors:** Lauren Dyer, Rachel Hardeman, Dovile Vilda, Katherine Theall, Maeve Wallace

**Affiliations:** 1 Mary Amelia Douglas-Whited Community Women’s Health Education Center, Department of Global Community Health and Behavioral Sciences, Tulane University School of Public Health and Tropical Medicine, 1440 Canal St., New Orleans, LA 70112 USA; 2 Division of Health Policy and Management, University of Minnesota School of Public Health, 420 Delaware St SE, Minneapolis, MN 55455 USA

**Keywords:** Adverse birth outcomes, Black women, Low birth weight, Mass incarceration, Maternal and child health, Preterm birth, Racial health disparities, Social epidemiology, social determinants

## Abstract

**Background:**

A growing body of evidence is beginning to highlight how mass incarceration shapes inequalities in population health. Non-Hispanic blacks are disproportionately affected by incarceration and criminal law enforcement, an enduring legacy of a racially-biased criminal justice system with broad health implications for black families and communities. Louisiana has consistently maintained one of the highest rates of black incarceration in the nation. Concurrently, large racial disparities in population health persist.

**Methods:**

We conducted a cross-sectional analysis of all births among non-Hispanic black women in Louisiana in 2014 to identify associations between parish-level (county equivalent) prevalence of jail incarceration within the black population and adverse birth outcomes (*N* = 23,954). We fit a log-Poisson model with generalized estimating equations to approximate the relative risk of preterm birth and low birth weight associated with an interquartile range increase in incarceration, controlling for confounders. In sensitivity analyses, we additionally adjusted for the parish-level index crime prevalence and analyzed regression models wherein white incarceration was used to predict the risk of adverse birth outcomes in order to quantify the degree to which mass incarceration may harm health above and beyond living in a high crime area.

**Results:**

There was a significant 3% higher risk of preterm birth among black women associated with an interquartile range increase in the parish-level incarceration prevalence of black individuals, independent of other factors. Adjusting for the prevalence of index crimes did not substantively change the results of the models.

**Conclusion:**

Due to the positive significant associations between the prevalence of black individuals incarcerated in Louisiana jails and estimated risk of preterm birth, mass incarceration may be an underlying cause of the persistent inequities in reproductive health outcomes experienced by black women in Louisiana. Not only are there economic and social impacts stemming from mass incarceration, but there may also be implications for population health and health inequities, including the persistence of racial disparities in preterm birth and low birth weight.

## Background

Adverse birth outcomes are a persistent public health issue. Preterm and low birth weight infants experience greater incidence of morbidities and mortality in their developmental stages as well as in adulthood. According to the Infant Mortality Statistics 2013 Period Linked Death dataset, 36.1% of infant deaths in the U.S. were related to preterm birth, and preterm infants had mortality rates that were 63% higher than for full term infants [1]. A study of a nationally representative sample of children (*n* = 74,565) also estimated that preterm birth was the underlying cause of more than 50% of cerebral palsy diagnoses, 15–20% of intellectual disabilities, and 10–15% of autism spectrum disorders and other learning disorders [2]. As these individuals mature, they additionally face higher systolic blood pressure given lower gestational age and lower birth weights, as well as low fasting plasma glucose when compared to individuals born at term and at a normal birth weight [3].

There are substantial and alarming racial disparities in reproductive health outcomes experienced by black women in the U.S. While the overall rate of preterm birth (< 37 weeks’ gestation) has steadily declined with improvements in healthcare access and quality, non-Hispanic black women continue to experience a 1.5 fold greater risk for preterm birth and double the risk of delivering a low birth weight infant (< 2500 g) compared to women who are non-Hispanic white, inequities that appear to be increasing [4]. Emerging research is beginning to explore how the unique combination of social, economic, and environmental factors experienced by black women in the U.S. may contribute to the continuation of reproductive health disparities [5].

Mass incarceration is a markedly racialized experience in the U.S. and an issue of growing concern for researchers interested in identifying the root causes of population health inequities. On an individual level, incarceration has been associated with greater prevalence of HIV/AIDS, diabetes, hypertension, pulmonary disorders, and mental illness when compared to those who have not experienced incarceration [6, 7]. At the aggregate level, mass incarceration disrupts the social and economic networks in a community and creates barriers to resources that are integral to achieving and maintaining population health [8]. Researcher Patricia Kelly contends that when communities endure the removal and reentry of large numbers of community residents via the carceral system, difficulties emerge in sustaining long-term relationships and linkages that are useful for solving everyday problems [9]. Because of the sensitivity of reproductive health to social and contextual determinants, communities affected by mass incarceration may also be vulnerable to adverse birth outcomes as related factors fluctuate [10].

In the U.S., jails are municipal or county-level detention facilities that are administered by local law enforcement agencies for the purposes of confining individuals who have been accused of or convicted for minor criminal offenses [11]. While state and federal prisons hold twice the number of people as jails, jails have almost 19 times the number of annual admissions and are perceived by criminal justice experts as the “front door” to mass incarceration [11]. As jail incarceration rates have continued to rise, there has been a disproportionate effect on black communities. Despite the fact that black individuals accounted for only 13% of the general U.S. population in 2014, they represented 35% of the total jail population [12]. The incarceration-related racial divide in Louisiana is significant, with incarceration rates that are nearly five times higher among blacks when compared to whites [13]. This inequity mirrors the entrenched racial gaps in adverse birth outcomes between black and white women in the state. In 2017, Louisiana preterm birth and low birth weight rates for non-Hispanic blacks were nearly twice the rates for non-Hispanic whites and were greater than the national average rates for all races combined [14].

The purpose of this analysis was to determine whether mass incarceration of black individuals in Louisiana is associated with adverse birth outcomes among black women. Recent studies have shown that indicators of structural racism – such as residential segregation and differential access to education, employment, income, and political power – share in the overall influence on racial health inequities as affected communities are denied equal access to the resources and opportunities needed to achieve health and well-being [15, 16]. We hypothesized that mass incarceration would also be an indicator of structural racism in Louisiana and that the risk for preterm birth and low birth weight would be higher among black women in parishes (county equivalent) with higher prevalence of jail incarceration within the black community relative to black women in parishes with lower prevalence.

## Methods

### Study population and outcome ascertainment

This analysis includes all birth records issued to non-Hispanic black women (black women, hereafter) in Louisiana in 2014 (*N* = 23,954), data accessed through the National Center for Health Statistics. We identified preterm births (those occurring < 37 weeks’ gestation) and low birth weight births (those < 2500 g) as our primary outcomes of interest. Birth records included a geographic identifier for maternal parish of residence for linkage to data on incarceration and other parish-level contextual factors. As all data were deidentified, this study was deemed exempt by Tulane University Institutional Review Board.

### Jail incarceration prevalence

Our primary exposure of interest was the parish-level jail incarceration prevalence among black individuals (count of black individuals age 16 to 64 in jail per 1000 black non-incarcerated residents). Jail data were isolated from prison data for this analysis in order to specifically estimate parish-level effects. Data were provided by the Vera Institute of Justice based on analysis of incarceration trend data from the Bureau of Justice Statistics, the Census of Jails, and the Annual Survey of Jails [17]. Since some counties are too small to have their own jail facilities, they may rely on nearby jurisdictions for detention purposes. Incarceration rate estimates for these counties are derived based on data from multi-jurisdictional jails and the sending county’s share of the combined resident population [18]. The incarceration prevalence was scaled by its interquartile range (IQR) for the purposes of estimating risk of preterm birth or low birth weight associated with an IQR increase in incarceration.

### Additional covariates

Other measures included in the analysis were individual-level covariates available on the birth record and known to be associated with adverse birth outcomes, including insurance type (Medicaid, private, and other, including self-pay), maternal age (< 20, 20–24, 25–29, > 30), parity (nulliparous, multiparous), and the Kotelchuck Index of Prenatal Care Adequacy based on the ratio of observed prenatal visits to the number that would be expected given dates of care initiation and delivery (< 50% = inadequate, 50–79% = intermediate, 80–109% = adequate, and 110% or more = adequate plus) [19, 20]. In order to isolate the association between the parish-level black incarceration prevalence and adverse birth outcomes within the black community independent of differences in socioeconomic contextual factors between parishes, additional parish-level covariates were derived from the 2014 U.S. Census American Community Survey, including the Gini Index, which measures population income inequality (0–1 scale), racial income inequality (absolute difference in white to black median household income), the 2010 U.S. Census classification of parishes by urbanicity (mostly urban, mostly rural, completely rural), and the percent of blacks with a bachelor’s degree or higher.

### Statistical analysis

We identified the distribution of maternal and parish-level characteristics within the total study population and stratified by outcome status. We mapped parish-level incidence of preterm birth, low birth weight, and jail incarceration across Louisiana in ArcGIS in order to visualize patterns and variation. Next, we fit modified Poisson regression models with generalized estimating equations to account for women clustered within parishes. The first model (Model A), estimated the relative risk (RR) and 95% confidence interval (CI) for preterm birth associated with an IQR increase in the prevalence of incarcerated black persons, adjusted for maternal and parish-level characteristics. Model B included the same covariates with low birth weight as the outcome.

Lastly, we conducted sensitivity analyses to test the robustness of our findings. Given the growing body of literature highlighting the association between area violence and adverse reproductive health outcomes [21–23], we sought to explore the degree to which high incarceration prevalence simply reflected greater exposure to crime (and its negative health consequences) as opposed to representing a broader marker of social inequality and structural determinants of health inequities for black women. Therefore, we fit the same models described above and additionally included the parish-level index crime prevalence to estimate associations with preterm birth (Model C) and low birth weight (Model D). The index crime prevalence is the count of crimes (criminal homicide, rape, robbery, aggravated assault, burglary, motor vehicle theft, larceny-theft, and arson) per 1000 residents, as described in the 2014 Uniform Crime Reporting System Report from the Federal Bureau of Investigation [24]. All data analyses were conducted in SAS 9.4. All maps used for this study were created using ArcGIS software by Esri. (ArcGIS® and ArcMap™ are the intellectual property of Esri and are used herein under license. Copyright© Esri. All rights reserved).

## Results

**Fig. 1:**
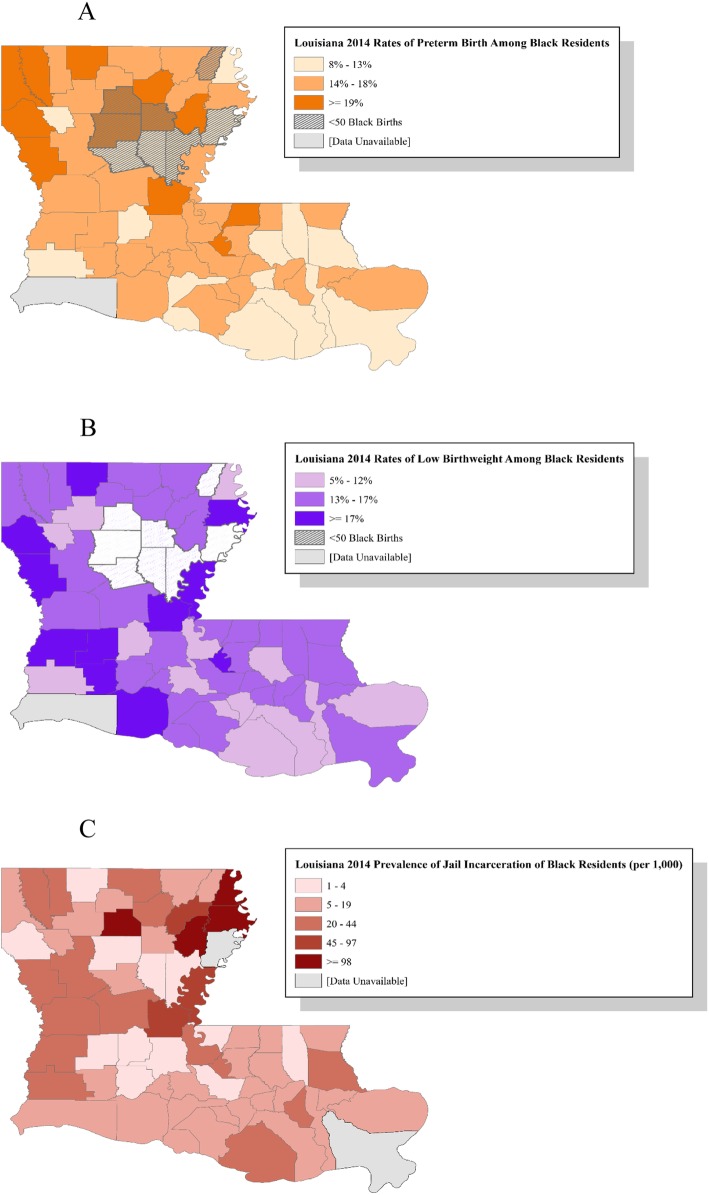
Distribution of percentage of adverse birth outcomes and incarceration prevalence, by parish, Louisiana 2014

A total of 23,954 births to black women were recorded in Louisiana in 2014. These births occurred across 63 of Louisiana’s 64 parishes; Cameron parish (which recorded no births to non-Hispanic black women that year) was not included in the analysis. Data on jail incarcerated populations by race were not available for Tensas and Plaquemines Parishes in 2014 and therefore births in these parishes were excluded. The final analytic sample with data on all covariates included 23,762 births in 61 parishes (99% of the total state births). Of these births, 15.34% were preterm (ranging from 8.47 to 25.53% across parishes; Fig. 1 panel a) and 14.99% were low birth weight (ranging from 5.08 to 33.33% across parishes; Fig. 1 panel b). The proportion of preterm birth was greater among those who were over 30 years old (18.75%), used other types of insurance (21.95%), had adequate plus prenatal care utilization (20.90%), were multiparous (16.55%), and were in mostly rural areas (16.49%) (Table 1). Among women who experienced low birth weight, prevalence was greater among women who were over 30 years old (16.80%), used other types of insurance (19.18%), had adequate plus prenatal care utilization (19.24%), were multiparous (15.11%), and were in mostly rural areas (16.22%) (Table 1).

**Table 1:** Characteristics of births to non-Hispanic black women in Louisiana, 2014 (*n* = 23,762)

	Total (%)	Preterm Birth (%)	Term Birth (%)	Low Birth Weight (%)	Normal Birth Weight (%)
	*N* = 23,762	*N* = 3644 (15.34)	*N* = 20,118 (84.66)	*N* = 3563 (14.99)	*N* = 20,199 (85.01)
Characteristic					
Maternal Age					
< 20	2569 (10.81)	344 (13.39)	2225 (86.61)	398 (15.49)	2171 (84.51)
20–24	8597 (36.18)	1188 (13.82)	7409 (86.18)	1255 (14.60)	7342 (85.40)
25–29	6767 (28.48)	1019 (15.06)	5748 (84.94)	931 (13.76)	5836 (86.24)
30+	5829 (24.53)	1334 (18.75)	4736 (81.25)	979 (16.80)	4850 (83.20)
Insurance					
Medicaid	19,961 (84.00)	2974 (14.90)	16,987 (85.10)	2969 (14.87)	16,992 (85.13)
Private	2972 (12.51)	488 (16.42)	2484 (83.58)	435 (14.64)	2537 (85.36)
Other	829 (3.49)	182 (21.95)	647 (78.05)	159 (19.18)	670 (80.82)
Prenatal Care Quality^a^					
Inadequate	5473 (23.03)	872 (15.93)	4601 (84.07)	846 (15.46)	4627 (84.54)
Intermediate	3226 (13.61)	409 (12.68)	2817 (87.32)	431 (13.36)	2795 (86.64)
Adequate	6011 (25.30)	471 (7.84)	5540 (92.16)	544 (9.05)	5467 (90.95)
Adequate +	9052 (38.09)	1892 (20.90)	7160 (79.10)	1742 (19.24)	7310 (80.76)
Parity					
Nulliparous	7859 (33.32)	1019 (12.97)	6840 (87.03)	1166 (14.84)	6693 (85.16)
Multiparous	15,730 (66.68)	2604 (16.55)	13,126 (83.45)	2377 (15.11)	13,353 (84.89)
Urbanicity					
Mostly Urban	20,493 (85.69)	3105 (15.15)	17,388 (84.85)	3034 (14.81)	17,459 (85.19)
Mostly Rural	2904 (12.22)	479 (16.49)	2425 (83.51)	471 (16.22)	2433 (83.78)
Completely Rural	365 (1.54)	60 (16.44)	305 (83.56)	58 (15.89)	307 (84.11)

^a^ Kotelchuck Adequacy of Prenatal Care Utilization Index

Incarceration prevalence varied across parishes in Louisiana from .76 per 1000 residents in Acadia Parish to 169.75 per 1000 residents in Winn Parish (Fig. 1 panel c). On average across births, the maternal parish incarceration rate was 16.58 per 1000 residents. The average Gini coefficient was .49, the average percentage of blacks with a bachelor’s degree or higher was 15% and the average absolute difference in median household income was $28,0009.69 (Table 2).

**Table 2:** Characteristics of Louisiana Parishes of Residence, 2014 (*n* = 63)

	Mean (IQR)
Black jail incarceration per 1000 residents	16.57 (21.27)
Absolute difference in white/black median household income (2014 inflation-adjusted US dollars)	28,009.69 (6600.34)
Gini index	.49 (.04)
Black educational attainment^a^	15 (.5)

^a^ Percent of the non-Hispanic black population age 25 and older with a bachelor’s or higher degree

Model A indicated that the relative risk for preterm birth was 3% higher per IQR increase in the prevalence of incarcerated black individuals after controlling for individual and contextual covariates (adjusted RR = 1.03; 95% CI: 1.00–1.06) (Table 3). Model B yielded an estimate of similar magnitude for low birth weight. The relative risk of low birth weight per IQR increase in the jail prevalence was 1.02 (95% CI: .99–1.05).

**Table 3:** Adjusted relative risks (RR) for preterm birth and low birth weight associated with black jail incarceration

Variable	Categories	Preterm Birth Model A RR (95% CI)^a^	*p* value	Low Birth Weight Model B RR (95% CI)	*p* value
Black jail incarceration prevalence (per IQR^b^ increase)		1.03 (1.00–1.06)	.0269	1.02 (.99–1.05)	.1148
Maternal age	< 20	1.06 (.92–1.21)	.4145	1.08 (.95–1.22)	.2344
	20–24	Reference			
	25–29	1.03 (.96–1.10)	.4537	.93 (.83–1.04)	.2043
	30+	1.22 (1.12–1.33)	<.0001	1.11 (1.00–1.23)	.0471
Insurance	Medicaid	.99 (.91–1.08)	.8186	1.08 (.98–1.19)	.1321
	Private	Reference			
	Other	1.43 (1.15–1.78)	.0013	1.38 (1.22–1.57)	<.0001
Prenatal care utilization index^c^	Inadequate	1.99 (1.77–2.23)	<.0001	1.69 (1.53–1.86)	<.0001
	Intermediate	1.58 (1.34–1.86)	<.0001	1.45 (1.29–1.64)	<.0001
	Adequate	Reference			
	Adequate +	2.65 (2.40–2.91)	<.0001	2.14 (1.95–2.35)	<.0001
Parity	Nulliparous	Reference			
	Multiparous	.82 (.78–.87)	<.0001	.99 (.92–1.07)	.8187
Urbanicity	Mostly Urban	Reference			
	Mostly Rural	1.11 (.94–1.31)	.2261	1.14 (1.01–1.29)	.0335
	Completely Rural	1.19 (.82–1.73)	.3684	1.16 (.88–1.54)	.2945
White – black median household income difference		.99 (.90–1.10)	.953	1.03 (.95–1.11)	.5088
Gini index		.79 (.16–3.89)	.7795	.59 (.24–1.48)	.268
Black educational attainment^d^		1.45 (.60–3.49)	.4088	1.70 (.69–4.16)	.2449

^a^ 95% Confidence Interval

^b^ IQR – Interquartile Range

^c^ Kotelchuck Adequacy of Prenatal Care Utilization Index

^d^ Percent of the non-Hispanic black population age 25 and older with a bachelor’s or higher degree

Results of the sensitivity analyses were consistent with our primary findings. Estimates of the association between incarceration prevalence and both preterm birth (Model C) and low birth weight (Model D) were essentially unchanged after the addition of the parish-level crime prevalence (Table 4). When additionally controlling for the index crime prevalence, women living in parishes with greater incarceration still experienced 3–4% higher risk for preterm birth or low birth weight relative to women in parishes with less incarceration (preterm birth RR = 1.04; 95% CI:1.01–1.07; low birth weight RR = 1.03; 95% CI:1.00–1.05.). In a supplemental sensitivity analysis, predicting the association between white incarceration prevalence while controlling for confounders did not yield significant results with the primary outcomes (preterm birth RR = 1.00; 95%CI: .99–1.01; low birth weight RR = .99; 95% CI: .99–1.00) (Table 5).

**Table 4:** Sensitivity analysis of violent crime in the association between birth outcomes and black jail incarceration

Variable	Categories	Preterm Birth Model C RR (95% CI)^a^	*p* value	Low Birth Weight Model D RR (95% CI)	*p* value
Black Jail Incarceration Prevalence (per IQR^b^ increase)		1.04 (1.01–1.07)	.0074	1.03 (1.00–1.05)	.0440
Maternal age	< 20	1.06 (.92–1.21)	.4281	1.08 (.95–1.22)	.2457
	20–24	Reference			
	25–29	1.03 (.96–1.10)	.432	.93 (.83–1.04)	.2087
	30+	1.22 (1.12–1.33)	<.0001	1.11 (1.00–1.23)	.0421
Insurance	Medicaid	.99 (.91–1.08)	.8537	1.08 (.98–1.19)	.1249
	Private	Reference			
	Other	1.43 (1.15–1.79)	.0012	1.38 (1.22–1.57)	<.0001
Prenatal care utilization index^c^	Inadequate	1.99 (1.78–2.24)	<.0001	1.69 (1.53–1.87)	<.0001
	Intermediate	1.56 (1.32–1.84)	<.0001	1.44 (1.28–1.63)	<.0001
	Adequate	Reference			
	Adequate +	2.66 (2.42–2.92)	<.0001	2.15 (1.96–2.36)	<.0001
Parity	Nulliparous	Reference			
	Multiparous	1.21 (1.15–1.27)	<.0001	1.01 (.93–1.09)	.8754
Urbanicity	Mostly Urban	Reference			
	Mostly Rural	1.18 (.99–1.39)	.0563	1.20 (1.06–1.36)	.0042
	Completely Rural	1.35 (.92–1.99)	.1284	1.29 (.97–1.72)	.0828
White – black median household income difference per 10,000		1.01 (.91–1.11)	.8853	1.05 (.97–1.13)	.2148
Gini		.34 (.05–2.19)	.2652	.31 (.11–.88)	.0277
Black educational Attainment^d^		1.52 (.64–3.59)	.3385	1.77 (.79–3.97)	.1667
Crime Rate (per IQR increase)		1.00 (1.00–1.01)	.0311	1.04 (.96–1.12)	.3711

^a^ 95% Confidence Interval

^b^ IQR – Interquartile Range

^c^ Kotelchuck Adequacy of Prenatal Care Utilization Index

^d^ Percent of the non-Hispanic black population age 25 and older with a bachelor’s or higher degree

**Table 5:** Sensitivity analysis of preterm birth and low birth weight risk associated with white jail incarceration

Variable	Categories	Preterm Birth Model E RR (95% CI)^a^	*p* value	Low Birth Weight Model F RR (95% CI)	*p* value
White Jail Incarceration Prevalence (per IQR increase)		1.00 (.99–1.01)	.8758	.99 (.99–.1.00)	.8564
Maternal age	< 20	1.06 (.93–1.21)	.4084	1.08 (.95–1.22)	.2307
	20–24	Reference			
	25–29	1.03 (.96–1.10)	.447	.93 (.83–1.04)	.2065
	30+	1.22 (1.12–1.33)	<.0001	1.11 (1.00–1.23)	.0484
Insurance	Medicaid	.99 (.91–1.08)	.816	1.08 (.98–1.19)	.1326
	Private	Reference			
	Other	1.44 (1.16–1.78)	.001	1.38 (1.22–1.57)	<.0001
Prenatal care utilization index^b^	Inadequate	1.99 (1.77–2.23)	<.0001	1.69 (1.53–1.86)	<.0001
	Intermediate	1.59 (1.35–1.87)	<.0001	1.46 (1.29–1.65)	<.0001
	Adequate	Reference			
	Adequate +	2.64 (2.39–2.91)	<.0001	2.14 (1.94–2.35)	<.0001
Parity	Nulliparous	Reference			
	Multiparous	.82 (.78–.87)	<.0001	.99 (.92–1.07)	.8032
Urbanicity	Mostly Urban	Reference			
	Mostly Rural	1.12 (.94–1.33)	.2028	1.15 (1.01–1.31)	.03
	Completely Rural	1.17 (.79–1.70)	.4298	1.15 (.86–1.52)	.3467
White – black median household income difference per 10,000		.99 (.89–1.09)	.8665	1.02 (.94–1.11)	.605
Gini		.79 (.16–3.93)	.7782	.60 (.24–1.51)	.279
Black educational Attainment^c^		1.23 (.49–3.03)	.6526	1.49 (.59–3.79)	.3914

^a^ 95% Confidence Interval

^b^ Kotelchuck Adequacy of Prenatal Care Utilization Index

^c^ Percent of the non-Hispanic black population age 25 and older with a bachelor’s or higher degree

## Discussion

This cross-sectional analysis of births among black women in Louisiana demonstrated that higher parish-level incarceration prevalence for black individuals were associated with significantly greater risks for preterm birth among parish residents. These results suggest that mass incarceration may – at least in part – contribute to the racial disparity in adverse birth outcomes in Louisiana. Moreover, this association was not explained by greater prevalence of crime in areas with high incarceration, as demonstrated in the sensitivity analysis, implying that prevalence of incarcerated black persons may be an important marker of structural inequality and a determinant of adverse birth outcomes among black women. Additional sensitivity analyses also demonstrated that white incarceration prevalence was not predictive of preterm birth or low birth weight among black women, suggesting that high incarceration overall may not be a proxy for a social condition impacting both blacks and whites, and is instead an indicator of the racism experienced by blacks.

While prior research has provided evidence of associations between mass incarceration and other public health outcomes [25], few studies have investigated the association between mass incarceration and preterm birth or low birth weight among black women in particular. The results of our analysis are generally in accordance with the minimal, but growing, body of literature on mass incarceration and reproductive health equity. For example, Wildeman’s analysis of Pregnancy Risk Assessment and Monitoring System linked data from 1990 to 2003 suggested that mass incarceration has a “substantial and significant” effect on black infant mortality [26]. In a separate analysis of health consequences of mass incarceration in an unbalanced panel dataset of developed democracies, including the U.S. 1981–2007 (*N* = 414), Wildeman concluded that with each 1 per 1000 increase in incarceration, there was a .25 per 1000 increase in the U.S. infant mortality rate [27]. Wildeman also found that for each 1 per 1000 increase in U.S. incarceration, there is an associated .29 (for U.S. males) and .37 (for U.S. females) decline in life expectancy years at birth for the sample, with these associations being highly significant at the .001 level. The present study and others suggest that mass incarceration may have long-term implications for population and perinatal health outcomes.

Chronic and psychosocial stress is another important risk factor for adverse birth outcomes, as demonstrated by a study in which preconception stress increased the odds for preterm birth by 19% and the odds of small-for-gestational-age birth by 14%, and consequentially increased the odds of infant mortality [28]. Mass incarceration is hypothesized to produce uniquely stressful situations because normal family functioning is disrupted and social and financial difficulties arise when helping formerly incarcerated relatives readjust to society [25]. For black women living in communities affected by mass incarceration, the increased exposure to these new stressors can lead to adverse birth outcomes. In a study of 5470 adults in the National Survey of American Life, Lee et al. estimated that women with an incarcerated family member experienced 1.44 times the odds of heart attack, stroke, diabetes, and obesity [29]. Lee et al. also postulate that women affected by incarceration are often already experiencing multiple stressors – such as poverty and community violence – even before this additional hardship. Another study found that women who lived in neighborhoods with greater levels of physical and social disorder - as well as violent crime - demonstrated increased levels of psychological distress and a higher risk for preterm birth [30]. The relationship between high incarceration prevalence and adverse birth outcomes may thus also be explained as a reaction to the stress experienced by pregnant individuals living in areas affected by mass incarceration.

With the increasing need for comprehensive interventions on the persistent gaps in perinatal outcomes for black women in the U.S., attention must be paid to factors that systematically disadvantage black communities, such as discriminatory arrest practices, unfair sentencing laws, and overuse of the bail system. For example, little is known about how disproportionally high contact with the criminal justice system among black individuals impacts population health and well-being. However, the findings of this study suggest that such a connection exists and that there are specific effects on black perinatal health. Inequities may also persist because race and gender are closely tied to resources like money, power, prestige, and social connectedness, and access to them is so integral to avoiding and minimizing disease that they should be considered potential fundamental causes of disease as well [31]. Tyan Parker Dominguez suggests that public health researchers and practitioners must shift focus from individual level risk factors to larger social forces that affect disease risk – including mass incarceration [32]. Dominguez also emphasizes that racism is a major contributor to health disparities since health outcomes are strongly influenced by socially constructed notions of race. In addition, there is growing interest in the use of the “life-course” approach to public health interventions to effectively eliminate reproductive health disparities by addressing early life disadvantages and life-long exposure to social determinants such as neighborhood poverty, crime, segregation, and racism [33].

This study has several limitations. Firstly, the cross-sectional nature of the data does not allow for conclusions to be drawn about causal pathways in the association between mass incarceration and adverse birth outcomes. Complete data on incarceration trends in Louisiana were only available up to 2014, so we were unable to analyze data that have become available following the 2017 adoption of comprehensive criminal justice reform in Louisiana which has resulted in a 2.9% decrease in state prison admissions of any type, and a 7.6% decrease in the total prison population [34]. Similarly, the data for birth outcomes- as well as for certain covariates, such as insurance type and prenatal care utilization - may have been affected by the expansion of Medicaid in Louisiana in 2016. As this reform was integral to changing the healthcare landscape of Louisiana, and since these covariates had strong effects on the preterm birth and low birth weight models, post-expansion data on these confounders should be analyzed as well. Future longitudinal research could explore how the association has changed over time and how criminal justice and healthcare reforms have impacted reproductive health equity in Louisiana. Additionally, while we controlled for several individual- and parish-level characteristics known to be associated with health outcomes, we acknowledge the possibility of residual confounders that may differ between women or parishes and contribute to preterm birth or low birth weight risk (racial residential segregation or other markers of structural racism such as racial inequality in employment and educational attainment, for example). There may be concerns about underreporting of crime in the FBI’s Uniform Crime Reporting System (the data source utilized for our measure of county-level crime prevalence), yielding a conservative estimate of both the true incidence and our measures of its association with adverse birth outcomes. Birth outcome incidence estimates for parishes with fewer than 50 black births should be interpreted with caution due to the small sample size. Lastly, since this analysis was conducted with black women in Louisiana the results may not be generalizable to other populations or women in other states.

## Conclusions

While scant research considers the indirect health consequences of mass incarceration, there is growing acceptance that it is a critical determinant of public health. Our findings support the notion that mass incarceration is a “social-structural” determinant of public health outcomes. Furthermore, given the disproportionate number of black people incarcerated in the U.S., mass incarceration is a critical indicator of structural inequity and must be acknowledged as a way that structural racism causes fundamental health inequity in our society [35], making Louisiana an important case study for understanding complex questions related to mass incarceration and reproductive health. To reduce the persistent inequities in adverse birth outcomes experienced by black women in the U.S., more research should focus on structural factors that contribute to poor health. Through collaborative research, mass incarceration can be viewed through a public health lens and partnerships between public health institutions and criminal justice officials can be utilized for monitoring incarceration data trends and formulating evidence-based interventions for improving the quality of life of those affected by mass incarceration and to reduce its overall social impact. Additionally, justice reforms can result in more fair and equitable sentencing practices, as has been seen in states who have adopted legislation to reduce sentences for minor offenses. In Louisiana, for example, recent bipartisan legislative support ended non-unanimous jury verdicts in felony trials in New Orleans, a decision expected to reduce unwarranted lengthy sentences and reduce racial bias in certain convictions [36]. Our findings compel the replication of these analyses in other states and counties with available data so that results may be compared and a more comprehensive understanding of the relationship between mass incarceration and birth outcome disparities among the black population can emerge. In particular, high prevalence of both incarceration and adverse birth outcomes across southern US states suggest the relevance of our hypothesis to their contexts as well.

## Data Availability

The data that support the findings of this study are available from the National Center for Health Statistics, but restrictions apply to the availability of these data, which were used under license for the current study, and so are not publicly available. Data are however available from the authors upon reasonable request and with permission of the National Center for Health Statistics.

## Abbreviations

CI: Confidence Interval
IQR: Interquartile range
RR: Relative Risk
